# Subjective quality of life and schizophrenia: results from a large cohort study based in Chinese primary care

**DOI:** 10.1186/s12888-024-05558-w

**Published:** 2024-01-31

**Authors:** Christine Migliorini, Carol Harvey, Cailan Hou, Shibin Wang, Fei Wang, Zhuo-Hui Huang

**Affiliations:** 1 Psychosocial Research Centre, Department of Psychiatry, The University of Melbourne, Grattan St, Parkville, Victoria, 3010 Australia; 2 Division of Mental Health, North West Area Mental Health, Melbourne, VIC Australia; 3 NorthWestern Mental Health, Royal Melbourne Hospital, Melbourne, VIC Australia; 4 Guangdong Mental Health Center, Guangdong Provincial People’s Hospital (Guangdong Academy of Medical Sciences, Southern Medical University, Huifu West Road Yuexiu District, Guangdong, China

**Keywords:** Quality of life, Psychological Well-being, Public Health, Schizophrenia, Cohort study, Self-Assessment, Adult

## Abstract

**Introduction:**

Much confusion exists between health-related QoL (HRQoL) scales and subjective QoL (SQoL) scales. One method to avoid confusion is use of a single question that asks *What is your quality of life?* or similar. This study explored the relationship between biopsychosocial factors and high SQoL, SQoL stability, and factors associated with improving SQoL.

**Method:**

We conducted a large cohort study of community-dwelling Chinese adults with schizophrenia, with two data points (2015–2016 (*N* = 742), 2017–2018 (*N* = 491)). Demographic and clinically related items and a comprehensive suite of published measures were collected. Direct logistic regressions were used to explore links between biopsychosocial factors and high SQoL and Improvement in SQoL across time.

**Results:**

Sample at Baseline: Male = 62.3%; *Med* age = 38.5 years; *Med* Age at illness onset = 24 years; SQoL *Mode* = neither poor nor good. Three independent variables predicted high SQoL at T1. Contemporary age and the presence of clinically relevant symptoms had a negative relationship with high SQoL; insight had a positive relationship with high SQoL. SQoL changed significantly across time with a modest effect size. Age at illness onset was the single independent variable linked to improving SQoL favoring being older at the time of illness onset.

**Discussion/Conclusions:**

SQoL can be high and changeable. While symptomology and illness insight may affect SQoL self-appraisals at single points in time, only age of illness onset was connected with improving SQoL. Thus, public health measures to delay illness onset are important. In addition, care about the distinction between HRQoL and SQoL in study design and choice of measures is necessary and will depend on the purpose and context.

## Introduction

Given the range and severity of symptomology experienced by individuals diagnosed with schizophrenia, symptoms and service use are prominent treatment and research interests [e.g., 1, 2–4]. Symptom mitigation and/or resolution is important but not at the expense of an individual’s life expectancy and quality of life both of which can be impacted by various medical treatments [5].

Health-related quality of life (HRQoL), a specialised-type of quality of life (QoL) measure, is increasingly accepted as an important outcome in health studies [6]. HRQoL measures are designed to examine the relative impact that health (or a health condition) has on an individual’s life qualities [7] and often used to monitor outcomes in clinical practice, clinical trials and population studies, especially for diseases with relapsing and remitting courses such as rheumatoid arthritis [8] and schizophrenia [9–11]. Encouraged by the WHO’s Constitutional principle of health being more than the absence of disease or infirmity [12] interest in more general QoL has also grown in health-related research including into persisting schizophrenia [e.g., 13, 14, 15].

While definitions of QoL abound, it is generally accepted that QoL consists of objective and subjective dimensions [16–19]. Typically, objective QoL is measured with indicators that you can count such as the size of one’s pay cheque or the size of one’s house. In contrast subjective QoL (SQoL) is a self-reported personal reflection and generally thought to come from an amalgamation of cognition and affect [20, 21]. Objective and subjective indicators are ordinarily poorly correlated [16, 19]. For instance, the average SQoL of waste pickers in South Africa was found to be higher than the national average [22] and the SQoL in individuals living with persisting serious disability such as tetraplegia can report living good or excellent lives [23] – as maintained by the *disability paradox* [24] whereby objective QoL is often low in individuals living with disability but low SQoL is much less likely.

Multiple factors have been linked to QoL in individuals living with schizophrenia and several meta-analyses have been completed. Davis and colleagues [25] found overall clinical insight to be negatively related to QoL i.e. higher QoL ratings linked with poorer insight. This relationship, however, was moderated by increased symptom severity. General psychopathology (e.g. depression and anxiety) was negatively linked with QoL and schizophrenia-specific symptoms (positive and/or negative symptoms) to be weakly linked at best [26]. A negative correlation between duration of untreated psychosis and QoL, and severity of symptoms and QoL exists in people experiencing first-episode psychosis [27]. Nevarez-Flores and colleagues [28] found global functioning to have a consistent positive association with QoL in people with psychotic disorders.

Each of the research teams who conducted the above meta-analyses, discussed QoL in generic terms. However, inspection of the included studies revealed that substantial proportions of studies used HRQoL measures. This is not unexpected since substantial heterogeneity abounds in the medical and psychological communities concerning QoL definitions, measurement tools and ways of reporting [29]. HRQoL studies are important. Arguably however, a person’s own appraisal of their life that we call SQoL (sometimes referred to as subjective wellbeing and/or satisfaction with life) can be as, if not more, important than an appraisal by a healthcare professional of the potentially negative impact of a health condition. The advantage of raising the profile of SQoL is that it encourages a more holistic appraisal of the individual’s life experiences and circumstances, putting the person ahead of the health condition. The issue is that HRQoL measures cannot examine beyond an absence of symptoms/lack of impact made by the health condition. Fundamentally HRQoL uses a deficit approach to measurement that is linked with, and contingent upon, the health condition [30] and consequently, cannot identify potential factors uniquely linked with good or high SQoL.

SQoL of individuals with schizophrenia has been the focus of other studies using a variety of measures. For example, a search of the Medline database using the parameters of: “subjective quality of life” and “schizophrenia” search terms, English language, and published between 2012 and November 2022, and after excluding citations that made only a brief reference to QoL [31–34], or reported a study protocol [35], yielded 71 citations. Of these, over a third of the studies used measures that were clearly HRQoL instruments such as the Schizophrenia Quality of Life Scale [36, 37], the Subjective Well-being under Neuroleptic Treatment Scale [e.g., 38, 39], and the health measures of MOS-36 [e.g., 40, 41] and the SF-12 [e.g., 42]. It seems there may be confusion between self-report as a data-gathering method and SQoL as a within person experience that is broader than the impact of a health condition.

One method to measure SQoL that cannot be confused with HRQoL is the use of a single question that asks: “*What is your quality of life?*”, “*How would you rate your quality of life*”, or similar. Single items to evaluate SQoL have been used to good effect in general population groups [e.g., 43, 44, 45] as well as with samples of adults living with schizophrenia [e.g., 46, 47–49]. In keeping with the *disability paradox*, in each of the samples the mean score sat just above the response scale midpoint suggesting that many individuals living with schizophrenia appraise their lives positively. However, there is a further unconsidered matter. In each instance, their analytical method assumed that their independent variables had a linear relationship with the SQoL dependent variable, implying that each factor is connected with both high and low SQoL in roughly equal measure. A more salutogenic approach is to ask, “What factors might be *uniquely* linked with high SQoL, despite exposure to the symptoms and consequences of living with schizophrenia”.

A recent large Chinese cohort study provided the opportunity to explore the relationship that biopsychosocial variables already linked with HRQoL including insight, psychopathology, duration of illness and symptomology, may have on high SQoL. The stability of SQoL could also be assessed. Four questions were posed.

1. What is the SQoL in Chinese adults living with schizophrenia?
2. Are there biopsychosocial variables connected with high SQoL?
3. How stable is high SQoL over time?
4. Is there a systematic difference between participants who reported their SQoL to be low at Time 1 (T1) and high at Time 2 (T2) compared to participants whose SQoL remained low?

## Method

### Study participants

Participants were adults (a) aged 18-years or older, (b) received a diagnosis of schizophrenia as per ICD-10 guidelines (c) fluent in Chinese - either Cantonese or Mandarin, (d) capable of understanding and completing the interview, and (e) registered for primary mental health services within one of the randomly chosen townships as per below. Both capacity and diagnosis were confirmed by one of the research psychiatrists during the clinical interview and by reviewing participants’ medical records. The exclusion criteria were history of significant head injury, seizures, cerebrovascular diseases, or other comorbid neurological disease.

A cohort study design with two time points was used to explore SQoL in adults with schizophrenia living in the county-level city of Luoding which is an underdeveloped and rural area in the south-west Guangdong province, southern China. This study used a random cluster sampling method to choose 21 of the 63 townships with primary mental health care services. All local patients with schizophrenia who were registered within these townships and managed within the Chinese National Psychiatric Management System (CNPMS) were approached. CNPMS was established to provide community follow-up management of people living with severe mental illness including schizophrenia; virtually all adults diagnosed with schizophrenia would be registered with CNPMS.

After eligibility was determined, written informed consent was obtained. Structured interviews with the adults and their caregivers, were undertaken by one of three research psychiatrists each of whom had 3-years or more of clinical and research experience. Data were collected within two 15-month periods (between time difference *M* = 24.7 months (*SD* = 3.80 months)): T1 Baseline in 2015 to 2016 (*N* = 742) and T2 Follow-up in 2017 to 2018 (*N* = 491), being a 66.2% response rate at follow-up. Those lost to follow-up did not differ significantly on any measure used in this study. The structured interviews encompassed socio-demographic and clinically related items and several validated measures which follow.

### Survey tool

SQoL was measured using the single broad question that prefaces the WHOQOL-BREF [50] whereby participants are asked *“How would you rate your quality of life?”* using a 5-point Likert scale anchored with very poor (score = 1) to very good (score = 5) response choices. As highlighted previously, using single items to measure SQoL has been demonstrated to be a convenient, valid, and reliable approach, both in the general population [51] and populations with a disability [52]. To meet the objectives of this study, SQoL was also dichotomised into High SQoL (scores 4 or 5) and Low SQoL (scores 1 to 3).

The Brief Psychiatric Rating Scale (BPRS) [53, 54] is a clinician administered scale consisting of 18 items that assess positive, negative, and affective symptoms in individuals who have a psychotic disorder. Item scores range from not present = 1 to extremely severe = 7, and 0 is entered if the item is not assessed. Scores are summed providing a summary total score from 18 to 126 with higher scores indicating the presence of more severe symptoms. The BPRS is a widely used scale purposely developed to evaluate schizophrenia-related symptoms over time [54]. The BPRS has been translated into Chinese [55, 56] and used to good effect (e.g. [57, 58]). Given this tool was positively skewed i.e., scores loaded to the left, the overall scores were grouped into No/minimal symptoms (scores 18–30) and Presence of clinically relevant symptoms (scores 31+) in accord with established cut-offs [e.g., 59, 60].

The Insight and Treatment Attitude Questionnaire (ITAQ) [61] is a clinician-administered scale comprising 11-items within two domains: understanding their illness (first 5 items) and understanding of their need for medication/hospitalisation (remaining 6-items). Item response scores are summed; each item is scored from 0 to 2 with higher scores indicating greater insight. The scale has been translated into Chinese with validity and reliability affirmed [62]. The distribution of scores for this tool was multimodal. Therefore, the overall scores were grouped into Low insight (scores 0–7), Medium insight (scores 8–13) and High insight (scores 14–22) for the regression analyses, based on those modal averages.

The Montgomery-Asberg Depression Rating Scale (MADRS) [63] is a clinician-administered 10-item instrument measuring the severity of depressive symptoms in adults with suspected depressive disorder, experienced over the previous 7-days. MADRS uses a 7-point Likert scale; each item ranges from 0 to 6 points. The scale is not meant to diagnose depression; however, it has high interrater reliability making it useful in clinical practice and research. The total score ranges from 0 to 60 points, and higher scores indicate more severe depressive symptoms. The MADRS has since been translated into Chinese with validity and reliability affirmed [64]. The overall MADRS-C scores were grouped into No to very mild symptoms of depression (scores 0–8), Mild level depression symptoms (scores 9–17), and Moderate to severe level depression symptoms (scores ≥ 18) based on established cut-offs [e.g., 65, 66] for descriptive purposes. This variable was partitioned into two groups (no/minimal symptoms (scores 0–8), and clinically relevant symptoms (scores 9+)) based on previous categorization and the distribution scores for the regression analyses.

The Sheehan Disability Scale (SDS) [67] is a self-report brief measure of functional impairment covering the three life areas of work/school, social and family life. Respondents nominate their perceived level of impairment using a 10-point visual analogue scale for each life area, with higher scores indicating higher levels of impairment. The three items can also be summed into a single dimensional measure of impairment. Given the high correlation across the subscales, only the total score was used in the regression analyses. The scale has demonstrated very good psychometric properties. The scale has since been translated into Chinese, with validity and reliability affirmed [68].

Socio-demographic and clinically related items collected at T1 baseline were also included in this study, being Sex (male/female), Contemporary Age (as at T1 baseline) Age at illness onset, Treatment satisfaction and Psychotropic polypharmacy, the last two items as follows. The participants were asked how satisfied they were with their current treatment. A 7-point Likert scale anchored with 1 = extremely dissatisfied to 7 extremely satisfied, was used. Medical records were audited for the type and range of psychotropic medication prescribed to participants. In this study, psychotropic medication included first and second-generation antipsychotic medication, antidepressant medication, benzodiazepines, anticholinergic medication, and mood stabilisers. This independent variable (IV) was partitioned into (a) none or one type of psychotropic medication, and (b) two or more types of psychotropic medications.

### Analyses

Question 1: Descriptive statistics were calculated.

Question 2: Direct logistic regression explores T1 biopsychosocial IVs that may predict high SQoL. The complete T1 Baseline sample was used.

Question 3: McNamar’s Tests examined the relative stability of high SQoL ratings across time. Interpretation of the strength of the relationships or effect size (*phi*) was based on Cohen’s recommendation of small = 0.10 to 0.29, medium = 0.30 to 0.49, and large = 5.0 to 1.0.

Question 4: Direct logistic regressions explored T1 biopsychosocial IVs that may predict individuals whose SQoL status improved, from low to high. The dependent variable (DV) was change of SQoL from T1 low to T2 high. Only those who rated their SQoL low at T1 and participated in both time points were used in this analysis.

*P* values less than 0.05 were deemed statistically significant across all statistical analyses. Effect sizes were calculated for statistically significant results. Diagnostic and Agreement Statistics package [DAG Statistics: 69] and IBM SPSS v27 were used to conduct the statistical analyses.

## Results

Most participants were male. Their median contemporary age was 38.5-years and median age at illness onset was 24-years. Just over half of the participants were taking no, or one type of, psychotropic medication. The validated scales BPRS-C, MADRS-C and SDS-C used in this study were positively skewed, especially the BPRS-C and MADRS-C: scores loaded to the left or lower end of the scales, indicating that their psychopathology symptoms and levels of impairment tended to be comparatively low. However, ITAQ-C total scores were multimodal suggesting a reasonable heterogeneity in participants’ insight into their illness and need for medication. See Table 1.

**Table 1:** Biopsychosocial descriptors of participants at Time 1

Variable	Subcategories	T1 Total initial sample Count (%)	T1 Subgroup who completed both timepoints Count (%)
Gender	Male	462 (62.3%)	313 (63.7%)
	Female	280 (37.7%)	178 (36.3%)
Psychotropic Polypharmacy			
	None or 1 type	414 (55.8%)	265 (54.0%)
	2 or more types	328 (44.2%)	226 (46.0%)
BPRS-C			
	No/minimal symptoms (scores 18–30)	566 (76.3%)	380 (77.4%)
	Clinically relevant symptoms present (scores 31+)	173 (23.7%)	111 (22.6%)
MADRS-C			
	Subclinical (no to very mild symptoms) (scores 0–8)	558 (75.2%)	376 (76.6%)
	Mild symptoms (scores 9–17)	150 (20.2%)	95 (19.3%)
	Moderate to Severe symptoms (scores 18+)	34 (4.6%)	20 (4.1%)
ITAQ-C			
	Poor insight (scores 0–7)	264 (35.6%)	174 (35.4%)
	Moderate insight (scores 8–13)	247 (33.3%)	162 (33.0%)
	Good insight (scores 14–22)	231 (31.1%)	155 (31.6%)
SQoL Rating			
	Very poor	9 (1.2%)	5 (1.0%)
	Poor	101 (13.6%)	69 (14.1%)
	Neither poor nor good	507 (68.3%)	338 (68.8%)
	Good	125 (16.8%)	79 (16.1%)
	Very good	---	---
		***Median (IQR)***	***Median (IQR)***
Contemporary Age (years)		38.5 (30.0–48.0)	40.0 (31.0–49.0)
Age of illness onset (years)		24.0 (19.0–30.0)	23.0 (20.0–30.0)
SDS-C	Work/School	4 (3–5)	4 (3–5)
SDS-C	Social	4 (3–5)	4 (3–5)
SDS-C	Family	4 (3–5)	4 (2–5)
Consumer satisfaction with current treatment		4 (4–5)	4 (4–5)

T1 – Time 1 Baseline; T2 – Time 2 Follow-up; BPRS-C – Brief Psychiatric Rating Scale – Chinese version; MADRS-C – Montgomery-Asberg Depression Rating Scale – Chinese version; ITAQ-C – Insight and Treatment Attitude Questionnaire – Chinese version, SDS-C – Sheehan Disability Scale – Chinese version

### Question 1

The most common SQoL rating was neither poor nor good. At T1, 125 participants (16.8%) rated their SQoL as good, and no-one rated their SQoL as very good. See Table 2.

**Table 2:** Distribution of SQoL response categories

		All T1 participants *(* *N* * = 742)*	*Participants of both T1 & T2 who completed SQoL item (* *n* * = 487)*	
Variable	Subcategories	T1 only Baseline Count (%)	T1 Baseline Count (%)	T2 Follow-up Count (%)
T1 SQoL Rating	Very poor	9 (1.2%)	5 (1.0%)	25 (5.1%)
	Poor	101 (13.6%)	68 (14.0%)	92 (18.9%)
	Neither poor nor good	507 (68.3%)	335 (68.8%)	138 (28.3%)
	Good	125 (16.8%)	79 (16.1%)	225 (46.2%)
	Very good	---	---	7 (1.4%)

### Question 2

Direct logistic regression was performed to assess the impact of several biopsychosocial factors on the likelihood that participants would report high SQoL. The model contained nine predictor variables (Sex, Contemporary age, Age of illness onset, BPRS-C, Psychotropic polypharmacy, MADRS-C (2 groups), ITAQ-C (3 groups), SDS-C total, and Treatment satisfaction). The high SQoL model was statistically significant with the full model able to distinguish between participants who reported high and not high SQoL, correctly classifying 83.5% of cases. Three cases (#273, #548, #607) were excluded as outliers (standardised residuals > 2.5), suggesting these cases were not well explained by the model. In each instance, the model predicted SQoL to be low when it was observed to be high.

See Table 3.

**Table 3:** Logistic regression predicting T1 high SQoL rating at Time 1 Baseline (excluding 3x outliers - cases #273, #548, #607)

IV Predictors	Subcategories	OR (95%CI)
Sex	Female	*Ref*
	Male	1.28 (0.83-2.00)
Contemporary Age		0.96 (0.93–0.98)
Age of illness onset		1.03 (1.00-1.06)
BPRS-C	No/minimal symptoms (scores 18–30)	*Ref*
	Presence of clinically relevant symptoms (scores 31+)	0.29 (0.13–0.65)
Psychotropic polypharmacy	None or 1 type	*Ref*
	2 + types	1.19 (0.79–1.79)
MADRS-C	No/minimal symptoms (scores 0–8)	*Ref*
	Presence of clinically relevant symptoms (scores 9+)	0.92 (0.51–1.65)
ITAQ-C	Low (scores 0–7)	*Ref*
	Medium (scores 8–13)	2.03 (1.13–3.63)
	High (scores 14–22)	2.65 (1.43–4.93)
SDS-C Total		0.99 (0.95–1.03)
Consumer treatment satisfaction		1.04 (0.82–1.32)
Constant		0.27
Significance of model	*Χ*^*2*^*(df, N) p*-value	61.455 (10, *N* = 739) *p* < 0.0005
Model percentage variance predicted	Between Cox & Snell R^2^ and Nagelkerke R^2^	8.0 − 13.5%
Percentage of cases correctly classified		83.5%

T1 – Time 1 Baseline; T2 – Time 2 Follow-up; IV – Independent variable; BPRS-C – Brief Psychiatric Rating Scale – Chinese version; ITAQ-C – Insight and Treatment Attitude Questionnaire – Chinese version, SDS-C – Sheehan Disability Scale – Chinese version

As shown in Table 3, three of the predictor variables made unique statistically significant contributions to the high SQoL model: Contemporary age, BPRS-C, and ITAQ-C. The strongest predictor of reporting high SQoL was the presence of clinically relevant symptoms, with an odds ratio of 0.29. Since this odds ratio is less than 1, this indicated that participants experiencing clinically relevant symptoms as measured by the BPRS-C were 3.4 times less likely to report high SQoL, controlling for all other factors in the model. The odds ratio of 0.96 for Contemporary age was less than 1, indicating that for every additional year of age, participants were 1.04 times less likely to report high SQoL. Moderate insight, as measured by ITAQ-C, approximately doubled the likelihood of reporting high SQoL (OR: 2.03) and having high insight more than two and a half times the likelihood (OR: 2.65), controlling for all other factors in the model.

### Question 3

Based on the subgroup of participants who contributed at both timepoints, SQoL remained the same for 53% of participants, leaving a substantial minority (47%) of participants changing high to low or low to high: *χ*^*2*^ = 101.78 (1, *n* = 487), *p* < 0.00005; the effect size was modest, *phi* = 0.049. See Table 4.

**Table 4:** Stability of SQoL rating

	T1 High SQoL	T1 Low SQoL	Total
T2 High SQoL	42	190	232
T2 Low SQoL	37	218	255
Total	79	408	487

The overall number of participants in this analysis is less than the total T2 sample as not all participants responded to all items

### Question 4

Based on the subgroup of participants who rated their SQoL as low at T1 and contributed at both timepoints, direct logistic regression was performed to assess the impact of several biopsychosocial factors on the likelihood that participants would report a change in QoL status (T1 Low to T2 High). The model contained niine predictor variables: Sex, Contemporary age, Age of illness onset, BPRS-C, Psychotropic polypharmacy, MADRS-C (2 groups), ITAQ-C (3 groups), SDS-C total and Treatment satisfaction. Results revealed only one predictor variable made a unique contribution to the model: Age at illness onset. However, the results for both the Omnibus Tests for Model Coefficients and Hosmer-Lemeshow Goodness of Fit Test, indicated the model to be a poor fit. Further checks revealed this was not due to data issues such as the undue influence of outliers or over/under dispersion. In the interests of parsimony and to potentially improve the model statistics, predictor variables that were non-significant in the T1 full sample model were removed and basic demographic variables were retained. This resulted in a model that contained 5 predictor variables: Sex, Contemporary age, Age of illness onset, BPRS-C (2 groups) and ITAQ-C (3 groups).

The resulting change in SQoL status model was statistically significant with the full model able to distinguish between participants who reported improvement and those who remained low, correctly classifying 58.5% of cases. See Table 5. One of the predictor variables made a unique statistically significant contribution to the impoving SQoL rating model - Age of illness onset (OR = 1.03). This indicated that each year older in age at illness onset increased by 3% the likelihood that SQoL changed from low to high, controlling for all other factors. See Table 5.

**Table 5:** Logistic regression model predicting SQoL rating improvement T1 Low to T2 High (*n* = 407)

IV Predictors	Subcategories	OR (95%CI)
Sex	Female	*Ref*
	Male	1.37 (0.90–2.09)
T1 Contemporary Age		1.00 (0.98–1.03)
T1 Age of illness onset		1.03 (1.00-1.06) ^a^
T1 BPRS-C	No/minimal symptoms	*Ref*
	Presence of clinically relevant symptoms	0.93 (0.57–1.52)
T1 ITAQ-C	Low (scores 0–7)	*Ref*
	Medium (scores 8–13)	1.47 (0.89–2.43)
	High (scores 14–22)	1.08 (0.64–1.83)
Constant		0.25
Significance of model	*Χ*^*2*^*(df, N*) *p*-value	13.203 (6, *N* = 408) *p* = 0.040
Model percentage variance predicted	Between Cox & Snell R^2^ and Nagelkerke R^2^	3.2-4.3%
Percentage of cases correctly classified		58.5%

T1 – Time 1 Baseline; IV – Independent variable; BPRS-C – Brief Psychiatric Rating Scale – Chinese version; ITAQ-C – Insight and Treatment Attitude Questionnaire – Chinese version

^a^ is 1.00 due to rounding down (is actually 1.001)

## Discussion

To date, few studies have considered high SQoL in community-dwelling populations with schizophrenia who are treated in primary care, nor explored factors that may be uniquely connected with high SQoL. At T1 this study found most participants perceived their SQoL to be either poor or middling. However, a modest subgroup did report their SQoL to be good, confirming the premiss that it is possible to consider one’s life to be good despite also living with a significant mental disorder such as schizophrenia. While rarely the focus of previous research, our result is in keeping with a few other studies [70–72]. Moreover, this study extends those findings to a non-western country, namely China.

Time had a significant relationship with SQoL. Multivariate analyses revealed older age of illness onset was significantly associated with improving SQoL. Immonen et al. [73] conducted a meta-analysis into the effect of age of illness onset and found earlier age of onset to negatively impact individuals in a range of long-term outcomes including more relapses, more hospitalisations, poorer social/occupational functioning and poorer global outcomes. It seems reasonable that being more mature before the onset of schizophrenia could advantage individuals. The extra time could provide more opportunity for individuals to accrue more material assets, grow supportive adult relationships, and develop a stronger sense of self before having to endure the onslaught of their first psychotic episode. Research has demonstrated that each of those factors– material resources, supportive relationships, stronger/more positive sense of self– reduce the impact of negative experiences such as onset of schizophrenia [e.g., 74, 75–77].

There is some evidence that while time is a linear construct, its impact may not be. Rotstein et al. [78] found the age of illness onset in males to have a curvilinear (smile-shaped) relationship with self-appraised SQoL connected with poorer SQoL outcomes (bottom of smile) for those aged around their mid- thirties at illness onset. The researchers acknowledge however, that their results seemed to run counter to previous literature. Contemporary age was negatively associated with SQoL at Time 1 in our study but not significantly associated with improved SQoL suggesting that extra time before illness onset might establish an advantage that is not easily overcome with extra time post-diagnosis. Further evidence exists demonstrating that the trajectory of illness presentation, treatment and functioning varies according to whether adolescent-or adult-onset, favouring adult-onset [79].

Clearly, time is still a conundrum needing to be explored more fully. Nevertheless, our overall findings underscore the need to defer illness onset as much as possible by public health measures. These might include campaigns to encourage young people to delay their first use of cannabis [80], as well as family psychoeducation and/or school-based programs [81, 82] in childhood and adolescence with the potential to decrease the overall risk of early onset, by preventing bullying [83].

Exploring the stability of SQoL, we found that nearly half of the cohort who participated at both timepoints and reported their SQoL to be low at baseline, reported an improvement in their SQoL over 24-months, shifting to the high category. This was statistically significant with a small effect. Several possible reasons for this change spring to mind. Perhaps care in the medical clinics improved for many of the participants, or the economy improved for many families thus affording larger economic reserves to help with participants’ care, or that being asked about one’s quality of life reminded participants to take better care of themselves, or participants whose SQoL improved were more likely to remain in the study. Lange et al. [15] also examined the trajectory of SQoL in older Dutch people with schizophrenia, finding nearly 56% of their participants had a clinically relevant change in SQoL across five years - some improved and some deteriorated. This suggests that living with chronic schizophrenia requires great effort by patients, families, and carers in the maintenance of SQoL; an effort that transcends cultural differences.

The presence of clinically relevant psychiatric symptoms was negatively associated with high T1 SQoL. These results are in keeping with previous research such as Eack and Newhills’ meta-analysis that examined the connection between psychiatric symptoms and QoL [26]. Their result was not unexpected given the high prevalence of HRQoL measurements included in their study samples. But our findings, using an unambiguous SQoL measure, also makes sense. It would be more challenging for most people to maintain high SQoL when concurrently experiencing significant symptoms. They are also in keeping with the pooled analysis by Priebe and colleagues [84] who found reduction in psychiatric symptoms was associated with improvements in SQoL. Severity of negative symptoms (diminished expression, amotivation) is also negatively associated with subjective well-being [70]. Once more, this study extends those findings to a non-western country.

Illness insight and medication compliance, measured by ITAQ, were significantly associated with high T1 SQoL in the cross-sectional multivariate analysis, with increased insight at least doubling the odds of reporting high SQoL. This seems reasonable if we surmise that a level of insight is needed to accept and comply with treatment for symptom remission, thus providing better opportunity for individuals to *live* their lives. But it does run counter to much of the literature discourse that features the insight paradox whereby increased insight is associated with decreased QoL thought to be moderated by feelings of hopelessness as featured in the meta-analysis by Davis et al. [25]. Yet, this too makes sense as most of the QoL measures used in their included studies administered HRQoL measures. Individuals who lack insight into their illness and consequent need for treatment/medication would be unlikely to acknowledge the negative impact of schizophrenia symptomology, when responding to items that measure the impact of illness on various qualities of life as per HRQoL scales.

Other independent variables including medication, and functional impairment, were not connected with high SQoL in our study, contrary to the evidence in the aforementioned meta-analyses and, arguably perhaps, expectations. Examination of each meta-analysis sample revealed substantial numbers of studies that used a HRQoL measure. HRQoL measures are designed to examine the relative impact that a given health condition has on various aspects (qualities) of an individual’s life so can be useful in treatment or service evaluations and research. But that is qualitatively different to assessing how an individual might appraise their own life as a whole and thereby might explain this discrepancy.

HRQoL measures are constructed within a deficit-based framework whereby the absence of impact by the given health condition symptom(s) is considered to be the equivalent of high QoL [30, 85]. For example, the Schizophrenia Quality of Life Scale [86] is a 30-item self-report measure that consists of three subscales: Psychosocial (15 items), Motivation and Energy (7 items), and Symptoms and side-effects (8 items. Symptoms in body or mind, are a sign of illness. Experiencing any health condition including schizophrenia means the presence of symptoms that will, inevitably, have a downward impact on a HRQoL scale. Unsurprisingly therefore, clinical, and function-related measures will more often demonstrate a significant relationship with a HRQoL scale whether clinician or patient completed.

A strength of this study was study design being a large cohort study thus increasing confidence in the generalisability of the results. The study was based in China which may limit generalisability to western settings. However, several of our results were in keeping with studies based in western countries suggesting the findings reflect features of common humanity rather than any given culture. This study used a single item SQoL scale to good effect. This had the advantage of removing any ambiguity in study topic. Single items also have advantages of reduced burden and costs – important considerations in any research. Nevertheless, a higher-order complex concept such as SQoL is better represented by a comprehensive multi-faceted scale and a wider scoring range.

## Conclusion

Of course, symptoms can and do change our own appraisals of SQoL but the impact in HRQoL is almost inevitable and, in SQoL, not so much, as found in the current study. In principle, even individuals living with significant disability such as tetraplegia [23] or living in extreme poverty [22], can report living a good life. HRQoL appraisals are important for health/treatment/service evaluation. But when examining an individual with any health condition it is important to remember they are just that – an individual *with* a health condition, and if one is interested in appraising overall QoL in general then using a general population SQoL measure is both appropriate and advisable. Consequently, care about the distinction between HRQoL and SQoL in study design and choice of measures is necessary and will depend on the purpose and context.

This study demonstrated that SQoL can change for individuals with schizophrenia. The implication for mental health services is that any individual living with schizophrenia and whose SQoL is currently good can change for the worse, and also importantly, vice versa. While symptomology and illness insight may affect SQoL self-appraisals in any given point in time, only (older) age of illness onset was connected with improving SQoL. Thus, it is important for public health departments to use activities such as campaigns to encourage young people (i.e. adolescents and young adults) to delay their first use of cannabis, and family psychoeducation and/or school-based programs in childhood and adolescence with the potential to decrease the overall risk of early onset e.g., anti-bullying programs, to delay the onset of schizophrenia.

## Data Availability

The original contributions presented in the study are included in the article, further inquiries can be directed to the Dr. Cailan Hou.
